# Educating minds with generative AI

**DOI:** 10.1038/s44271-026-00522-8

**Published:** 2026-09-14

**Authors:** Laura Desiree Di Paolo, Andy Clark, Thomas Wachter

**Affiliations:** 1 Department of Engineering and Informatics, University of Sussex, Sussex, UK; 2 ACoRNS, School of Psychology, University of Sussex, Sussex, UK; 3 Department of Philosophy, The University of Sussex, Brighton, UK; 4 National Center for Artificial Intelligence (CENIA), Santiago, Chile

**Keywords:** Education, Complex networks, Education

## Abstract

Generative artificial intelligence (GenAI) is rapidly entering education, framed as a tool for efficiency and personalization. In this Perspective, we argue this obscures a deeper transformation. Schools are cognitive ecologies in which tools and social practices actively shape learning. GenAI restructures this ecology, redistributing epistemic labour, consolidating pedagogical functions, and reorganizing how knowledge is accessed, produced, and evaluated. Unlike most educational technologies, it is active, persistent, and generalist. We identify two enduring misalignments: a pedagogical gap between learning sciences and AI design, and a goal gap between measurable performance and developmental aims. Both gaps reflect logics already embedded within existing educational systems organized around efficiency, standardization, and control. GenAI does not introduce but risks entrenching and amplifying these gaps. Rather than accommodating GenAI through technical adjustments, we propose treating it as a diagnostic opportunity to redesign schooling for embodied, collaborative, and distinctly human forms of learning.

## Abstract (web summary)

Generative artificial intelligence (GenAI) is rapidly entering education, framed as a tool for efficiency and personalization. This Perspective argues that this framing of GenAI obscures a deeper transformation.

## Introduction

Education derives from the Latin *educare*: to nourish, to cultivate, to draw out. As such, education is never a neutral enterprise: every educational system is organised around aims, whether explicitly articulated or tacitly embedded in its practices and structures^1,2^. The question of what education is *for* is therefore foundational, not optional. Importantly, these aims are never realised in the abstract or in a vacuum. They are enacted, stabilised, and transmitted through the concrete arrangements of educational practice, most notably through the physical tools, technologies, and social practices that structure learning environments.

Moreover, formal education has never operated independently of technology. From books, blackboards, and abacuses to calculators and computers; from individual desks to open-space classrooms and digital e-learning platforms, schooling has always relied on material artefacts to scaffold learning. These artefacts do not merely convey information; they become functionally integrated into cognitive activity, shaping how learners attend, reason, remember, and act. In this sense, the skills and forms of knowledge cultivated in schools are inseparable from the tools and technologies through which they are developed (for an account of hybrid cognition^3^). Thus, even if not always explicitly acknowledged, schools have always been hybrid, multi-technological cognitive ecologies, in which tools, materials, norms, and routines jointly structure learners’ agency and epistemic habits over development (see, e.g., refs. ^4–6^.

However, the recent advances in artificial intelligence (AI), particularly the emergence of large language models (LLMs) and the widespread adoption of generative AI (GenAI), mark a significant inflexion point in the long-standing relationship between education and technology, offering new possibilities but also posing tensions for educational systems^7^. Praised for their advanced linguistic capabilities, flexibility, and the ability to enable low-friction natural language interaction across a wide range of domains, these systems are increasingly promoted as tools that will deliver long-promised educational benefits to teachers, students, and institutions alike^8^. As a result, they have become a central focus of educational policy debates, institutional initiatives, and classroom experimentation^9^. Particularly, GenAI has been presented as *the* technical innovation that will overcome the limitations of earlier educational technologies, offering new possibilities for the generation of personalised learning materials, enhanced engagement and interaction, and teaching experiences tailored to individual needs^10,11^.

At the same time, ethical, social, ecological, and cognitive concerns are increasingly being raised in response to the uncritical adoption of these technologies^12,13^. In education, one line of criticism argues that AI systems, often portrayed as ready-made technical solutions to educational problems, are introduced without sustained reflection on the pedagogical practices and institutional structures into which they are embedded^9^. Importantly, these practices and structures are not neutral backdrops but actively organise schooling; they will therefore condition how GenAI systems are implemented and the directions in which teaching, learning, and development are steered.

While some studies suggest that pedagogically-aligned implementations of AI can support learning (e.g., refs. ^14–16^), emerging evidence indicates that intensive and unguided use of LLM-based systems may erode precisely those (meta)cognitive capacities (e.g., critical thinking, epistemic ownership) that underpin effective learning and that education is expected to cultivate^17^. This raises significant questions about the impact of such technologies on students’ cognitive development, even in cases where they are adopted with the explicit aim of enhancing it^18^. These findings, however, remain preliminary, and they are situated within a broader and as yet unresolved debate over whether AI systems primarily function as instruments of cognitive enhancement or, conversely, as drivers of cognitive deskilling (e.g., refs. ^19,20^). It is against this backdrop of empirical uncertainty that the absence of sustained reflection on pedagogical and institutional conditions of deployment becomes especially consequential^21–23^ (see also ref. ^20^).

We aim to move beyond this tension and to address what we take to be a more fundamental issue in thinking about the integration of GenAI into education. Like other technologies, but in a far more radical way, GenAI cannot be understood merely as an additional tool available to educational practice. Rather, we propose conceptualising these systems as powerful cognitive artefacts (for definitions and examples, see refs. ^24–26^) whose educational significance cannot be adequately assessed solely in terms of individual gains or losses.

Instead, their impact must be examined in terms of whether and how they operate within the cognitive ecology of schooling, redistributing epistemic labour among learners, teachers, and technologies, and reshaping the practices, norms, and environments in which learning takes place^27,28^. It is of particular relevance that GenAI systems increasingly consolidate core teaching and learning functions (e.g., feedback, assessment, explanation, content generation, tutoring) within a single technological interface^10^, transforming how knowledge is accessed, produced, and valued. Likewise, it must be noted that this reorganisation does not occur in a pedagogical vacuum, but instead intersects with and amplifies educational structures already in place, some of which have become increasingly misaligned with contemporary understandings of learning, development, and cognition^29,30^. In this sense, GenAI should be understood not primarily as a disruptive force in education, but as an enhancer and intensifier of limitations *already* embedded in inherited educational practices.

We contend that, even more clearly than during the COVID-19 pandemic (e.g., refs. ^31–33^), the current introduction of GenAI, while undoubtedly introducing new risks, creates a critical and timely opportunity to reassess the aims, structures, technologies, ethics, and practices of schooling. This diagnosis calls for reframing the problem: away from efforts to accommodate AI within inherited schooling through technical fixes (e.g., adapting models to established curricula or classroom routines), and toward reimagining education for near futures in which technologies play an increasingly constitutive role in how learning is organised, both inside and beyond the classroom. If AI is becoming part of the infrastructure of schooling, it cannot be assessed primarily at the level of individual models or their isolated impacts. Instead, research and practice should examine how AI systems interact with, amplify, and reorganise existing educational ecologies and pedagogical arrangements, and how these ecologies, in turn, shape what AI systems actually come to do in practice. This includes analysing how entrenched structures - such as the centrality of testing in everyday school routines, or the framing of teaching as the delivery of small, discrete *pebbles* of knowledge to students - configure the roles AI is permitted or expected to assume.

This shift demands timely attention, given the stakes involved. On the one hand, the uncritical introduction of GenAI systems risks reinforcing outdated pedagogical assumptions under the guise of innovation, as evidenced by the prevalence of LLM-based chatbots designed around individualised, screen-bound, and largely disembodied modes of learning, albeit (at least) personalised^34–37^. On the other hand, the longer-term consequences of consolidating functions and practices within such disembodied systems for students’ socio-emotional and cognitive development remain largely unknown. Integrating current keyboard-and-screen-based AI systems into existing educational ecologies may do little more than scale prevailing instructional models; yet it may also produce stronger, less predictable effects on social competences and well-being that exceed present understanding (see, e.g., refs. ^38–40^). Therefore, instead of defaulting to incremental integration, this moment should be taken as an opportunity to critically reconfigure pedagogical practices in line with contemporary research on learning and development, which has consistently demonstrated the benefits of those educational approaches that foreground movement, embodied interaction, and engagement with materials, environments, and others (e.g., refs. ^41–45^). Ultimately, integrating AI should not be an end in itself, but part of a broader (re)configuration of educational technologies guided by sound pedagogical approaches.

We begin by examining educational settings as cognitive ecologies through the lens of the 4E account of cognition (2). We then identify three challenges in the uncritical introduction of GenAI into education: a *Pedagogical Gap* (2.1), a *Goal Gap* (2.2), and the risks and potentials of AI hallucinations and confabulation (2.3). We conclude by proposing a catalytic role for AI within a multitechnological ecological approach, one in which AI becomes a tool, not the tool, for rethinking what education is for.

## Education as epistemic infrastructure

One way to begin with is by adopting a different understanding of cognition, one that does not treat learning and other cognitive functions as exclusively internal mental processes and mere outcomes of brain activity, but emerging from dynamic interactions between the agent and its bodily, social, and material environment. This perspective is commonly described as the “4E” account of cognition, according to which the mind is embodied, embedded, enacted, and, in some cases, extended (e.g., refs. ^46,47^).

Applying this framework to education requires reconsidering what learning environments and technologies are: not neutral settings or mere conduits for knowledge transmission, but hybrid, multi-technological, and deeply epistemic cognitive ecologies. Formal educational systems are relatively stable, institutionally mediated, culturally structured, and explicitly designed to shape cognition over developmental timescales. By organising the environments, practices, and norms through which cognitive capacities are cultivated, particularly in early development, education does not simply support cognition: it actively structures how cognition itself develops across individuals and generations (see, e.g., refs. ^48–50^). Education could be interpreted not only as an application of the 4E account of cognition, but as its extension: a fifth dimension for which cognition is also *educated*.

In this light, educational technologies are never neutral tools. By shaping which forms of engagement are foregrounded and which epistemic practices are normalised, they function as scaffolds that structure how future generations of students learn and develop. This means analysing pedagogical impacts not only in terms of isolated learning outcomes for individual students, but mostly in terms of how learning environments are organised at scale, how agency is distributed, and which forms of knowledge are afforded - or rendered less visible - over time. Under this point of view, integrating GenAI in educational settings does not mean implementing external aids, but transforming the very conditions under which cognition is enacted in schools.

This shift is best understood by conceptualising GenAI as an epistemic infrastructure and as a transformative element within existing ones^51^. Epistemic infrastructures, whether physical or institutional (e.g., libraries, laboratories, hospitals, or ritual practices), do more than provide access to knowledge: they organise how knowledge is produced, circulated, evaluated, and legitimised. By structuring epistemic activities and regulating epistemic effort, they stabilise the norms that define what counts as competent participation in cultural practices^52,53^. When introduced and institutionalised into education, GenAI systems might effortlessly assume this infrastructural role. AI-powered teaching management systems (e.g., *Toddle*, *ManageBac*) deliberately consolidate core functions (curriculum planning, instructional support, learner assessment, and progress reporting) within a unified, algorithmically mediated environment, imposing a mandatory participation structure on all educational actors, including teachers, administrators, secretaries, principals, and parents, who must adapt their practices to the system’s operational logic regardless of individual preference or unaligned with prior workflow. Modifying standards of explanation and assessment, regulating teaching and learning activities, they reshape what counts as legitimate participation in the educational system itself^54^ (see also ref. ^55^).

Most educational tools primarily mediate access to information or scaffold how content is presented. Whether books, overhead slides, or physical and digital games, they support human activity without acting on the user’s behalf: their functions are typically specialised, temporally bounded, and fundamentally passive. A book, for example, is used intermittently, contains fixed and often partial information, and cannot adapt to changing contexts or tasks. It is worth noting, however, that there has always been some educational materials and environments deliberately designed not to support a single specialised function, but to enable multiple operations almost simultaneously. Comenius’s *Orbis Pictus* (1658) was conceived to teach vocabulary, language, and world knowledge through the integrated use of image and text within a single volume^56^. Papert’s Logo programming environment was designed to merge mathematical reasoning, problem-solving, and epistemic reflection into a unified practice, in which debugging code and developing conceptual understanding were not separate activities but facets of the same process^57–59^. Abbott’s *Flatland* (1884), while not intentionally a pedagogical instrument, might operate as a lesson^60^ and, simultaneously, inform about a historical period and elicit a critical representation of it. Even purpose-built multisensory artefacts, such as the Montessori ‘Stamp Game’ for mathematics or ‘Puzzle Maps’ for geography, explicitly designed to be self-correcting and to engage multiple cognitive functions and sensory channels simultaneously, remain bounded in their affordances (see e.g., refs. ^61–63^. A physical globe cannot autonomously provide greater detail about a particular country or city on demand; the *Orbis Pictus* cannot answer a question it does not contain; the Logo environment requires a teacher or peer to provide affective scaffolding; *Flatland* cannot adapt its geometry lesson to the reader’s understanding, nor elaborate its social critique beyond what the text can afford. In each of these cases, multifunctionality was deliberate. Yet, each artefact carries a ceiling determined by its materiality and its author’s intent, and the user must frequently move outside the object itself to seek elaboration, clarification, or assessment.

AI-based conversational systems operate on a qualitatively different register, functioning as active, or at least semi-active, participants in epistemic processes. Although they do not possess human-like agency, nor access to the sensory channels that some other educational devices engage, they can plan and execute sequences of actions on a user’s behalf with limited supervision^64^, enabling increasing degrees of functional autonomy. Continuously updated and reconfigured after deployment, they become enduring and highly adaptable components of educational environments rather than static tools. This combination of autonomy and adaptability allows GenAI to perform epistemic functions that, until recently, were largely reserved for human cognition or required constant oversight, including problem framing, explanation generation, evaluation, and the selection and organisation of relevant information, such as checking test responses against an answer key^65–67^, as well as forms of mental and emotional support in educational contexts and beyond^68–74^. Within a single interface, they can subsume functions ranging from instruction and error correction to adaptive scaffolding and proto-emotional support such as encouragement, dynamically calibrating to the user’s level of knowledge and requiring comparatively little prior competence to access^75^. Their adoption thus marks a qualitative shift: from tools that support human activity while rarely combining multiple properties simultaneously (e.g., refs. ^38,76^), to systems that increasingly participate in, and may autonomously reorganise the epistemic processes themselves. This is not a question of moral hierarchy between technological paradigms. It is, however, necessary to acknowledge that the convergence of multiple (pedagogical) functions within a single system substantially amplifies both the possibilities and the associated risks, a scaling effect that is qualitatively distinct in the case of AI technologies.

GenAI’s linguistic fluency, apparent generality, and low interactional cost facilitate its rapid integration into everyday educational practice, even in the absence of sustained pedagogical framing or oversight (e.g., ref. ^77^). As a result, its influence on learning is both pervasive and difficult to delimit, extending beyond support to discrete teaching and learning tasks (such as grading assessments and correcting homework) to the shaping of routines, expectations, and norms through which educational activities are organised (such as developing alternative grading methodologies or homework schedules). From the perspective of hybrid and extended accounts of cognition, learning activities that were previously distributed across various elements (e.g., students, teachers, peers, texts, and different material artefacts) risk becoming increasingly delegated to, and concentrated within a single technological system. Given its growing functional autonomy, GenAI may come to direct processes of knowledge formation, largely unsupervised, if operating simultaneously as an informational source and systemic intermediary that can substitute for practices such as reasoning, synthesis, and evaluative judgment.

It is also important to remember that these GenAI enter educational settings not as a neutral instrument, but as a co-agent reflecting the values and constraints of the stakeholders involved in development and optimisation processes, and capable of actively, and in some cases autonomously, shaping how learning and knowing are enacted. For instance, despite the multiple efforts to mitigate prejudices, GenAI systems still embed demographic^78^, gender^79^, religious^80^, linguistic^81^, and even pedagogical biases^82^, arising from the characteristics of their training data, supervision regimes, and optimisation processes^12^. Ongoing post-deployment modifications, including fine-tuning, alignment adjustments, and policy-driven constraints, can substantially alter model behaviour over time, determining which kinds of content are generated, emphasised, or suppressed, and reflecting shifting normative and institutional priorities^83^. Recent public debates surrounding the post-release modification of commercial language models (e.g., alignment changes in the model *Grok*) illustrates how such systems can be continuously adjusted to reflect evolving organisational or political commitments, underscoring that GenAI systems do not merely reproduce biases inherited from training data, but actively incorporate value judgements introduced during deployment and governance. As a result, they systematically privilege particular forms of explanation, expression, and problem-solving while marginalising others, whether because certain perspectives are underrepresented in training corpora^84^ or because selective design choices are introduced during training and deployment. The associated risks range from the homogenisation of written production and reasoning patterns^85^ to the disproportionate misclassification of non-English students’ work as AI-generated by automated detection systems^86^ and to the implicit promotion of specific pedagogical models within lesson-planning and instructional design tools^82^.

In educational contexts, and in the absence of sustained and explicit reflection on pedagogical infrastructures and methodologies, GenAI is likely to amplify values already dominant, such as performance, standardisation, optimisation, and error avoidance^8,87^. As a consequence, educational practices risk drifting toward what is easiest to generate, evaluate, and scale, thereby inadvertently enabling a structural shift in the sources of epistemic authority that shape cultural practices and public knowledge, a process referred to as *epistemic substitution*^22,51,88^.

On the other hand, if cognition is understood as fundamentally intertwined with material and technological arrangements, and GenAI is acknowledged as an epistemic infrastructure, one that enables certain forms of knowing while constraining others, the central question shifts from ‘how to implement’ to ‘how to align with pedagogical values’. The issue is no longer simply how to deploy these systems, but how they might reshape educational cognitive ecologies, which forms of agency they distribute, which epistemic habits they cultivate or inhibit, and how these transformations correspond to contemporary theories of learning and the normative aims of education. Viewed through these lenses, we identify two persistent misalignments in current approaches, which we term the *pedagogical gap* and the *goal gap*.

### Pedagogical gap

The *pedagogical* gap, situated within a broader disconnection between learning science and technology design^89^, captures the misalignment between contemporary 4E-informed understandings of learning and the pedagogical assumptions implicitly encoded in current AI systems. This misalignment, however, should not be read as an indictment of AI as such. Current GenAI systems are largely designed and deployed within, and therefore reproduce, a pedagogical logic that operationalises learning in predominantly disembodied, abstract, and linguistic terms^34,90^. A logic that predates AI and remains pervasive across mainstream educational practice. The friction with research emphasising learning as an active, situated, embodied, and socially mediated process (e.g., refs. ^42,44,91–100^) is therefore not an intrinsic property of AI systems, but rather a consequence of the assumptions baked into their design. To the extent that a pedagogical gap exists, it reflects the broader dominance of disembodied pedagogical frameworks in educational technology more generally, of which current AI implementations are one instantiation.

This becomes concrete when considering the contrast between 4E-inspired pedagogical approaches and the design of current AI systems. Where 4E-informed pedagogies promote learning practices grounded in movement, active participation, and collaboration (e.g., ref. ^101^), in which students engage in perceptual, sensory, and motor activities to deepen their understanding of ideas and concepts^102^, current educational AI systems continue to be conceived and realised as individualised, screen- and keyboard-based assistants^35,87^, structurally limiting opportunities for bodily engagement, social interaction, and interpersonal understanding. The theoretical assumptions underlying these systems rely on models of cognition that make little reference to the learner’s body or situated activity, treating learners primarily as recipients of symbolic representations rather than as embodied agents engaged in relational and exploratory sense-making^36^. This misalignment extends beyond AI systems deployed as tutors. AI-based lesson-planning and instructional design tools—now widely adopted in schools—also embed narrow pedagogical assumptions that constrain students’ opportunities to make choices, act meaningfully in their learning, and engage collaboratively with others^82^. Developed within historically directive and teacher-centred models of instruction, these systems tend to reproduce highly structured lesson formats in which agency is attributed primarily to the instructor, whether human or artificial, rather than supporting exploratory, collaborative, or embodied forms of learning. By neglecting the sensorimotor and social foundations of human learning, such designs might render these technologies pedagogically ineffective and, in some cases, potentially counterproductive, particularly in absence of teacher oversight or explicit pedagogical guidelines^17^. They might perpetuate limited, discriminatory, and decontextualised conceptions of the human mind^36^, overlooking diverse cognitive profiles, learning preferences, and the environmental supports required by neurodivergent learners^103^.

It is worth noting that some implementations have begun to explore alternative pathways aimed at closing this gap, embedding GenAI within mixed reality environments to afford more active and cognitively engaging learning experiences^104,105^, or integrating it within embodied, collaborative pedagogical frameworks to promote more inclusive outcomes, including for students with special educational needs^106^. While promising, however, these cases remain exceptions, and their capacity to produce significant or lasting change may be structurally constrained. In our view, the gap reflects a persistent tension between “progressive” accounts of learning and more “conservative” institutional and technological arrangements, inherited physical spaces, tools, and social structures, that continue to shape schooling practices and resist viewing educational institutions as multi-technological cognitive ecologies. When GenAI is introduced into environments governed by such arrangements, it risks stabilising rather than challenging outdated pedagogical assumptions, regardless of the intentions behind its design. Closing the gap, therefore, requires more than technical innovation: it demands broader pedagogical, institutional, and cognitive reflection on the conditions under which any technology is deployed, a point well captured by Weidlich and colleagues’ reminder that “the efficacy of learning is determined by the instructional method, not the medium through which it is delivered” (ref. ^107^: p.2).

#### Interfaces

The pedagogical gap is particularly visible at the level of ‘interface design’ through which GenAI is currently introduced into educational contexts, and the educational methodologies it elicits. Most GenAI-based tools rely on a narrow interaction paradigm: learners formulate queries, the system generates responses, and the exchange either ends or iterates along the same linear trajectory. This prompt–response structure is constructed around the student’s primary task to learn to ask the “right” question (by tweaking the prompt), while the system’s role is to deliver a coherent and authoritative answer. Empirical evidence suggests that, within this interface configuration, students lacking clear guidance or sufficient AI-related knowledge rarely move beyond prompt tuning, resulting in interactions of limited educational value and increased reliance on system outputs^108^. Despite affording on-demand, individualised explanation, such AI tutors or learning companions ultimately mirror instructionist pedagogies: decomposing knowledge into discrete units and evaluating progress through correctness and fluency^109^, or by means of extrinsic methods (e.g., ref. ^110^, but see ref. ^111^).

Moreover, in the absence of sustained pedagogical reflection, the introduction of GenAI risks collapsing^112–116^ the distributed organisation of teaching and learning that even the most conservative schools typically sustain across people, artefacts, and temporal rhythms^117,118^. In a conventional classroom, for instance, teachers question and explain from the front-room; peers challenge, negotiate, and compare answers - sometimes informally whispering at the back - thereby generating productive social and epistemic friction; materials introduce resistance through missing notes, broken pencils, or incomplete drafts; scheduled assessments impose delay, revision, and reflection; and even the school bell structures attention and behaviour, establishing shared temporal and emotional routines that organise when and how learning occurs.

By contrast, GenAI systems increasingly compress multiple pedagogical functions into a seamless interface. Explanation, feedback, evaluation, and content generation are delivered instantaneously within the same interactional loop, typically through chat-based or voice-based exchanges, smoothing over the frictions and ambiguities that play a pivotal role in learning^119,120^. This design logic rests on assumptions that construe cognition as primarily internal, linguistic, and individual: not an intrinsic property of AI, but one that paradoxically prevents it from realising its full educational potential. This becomes evident when considering what current implementations foreclose.

Although immediate feedback might be beneficial (e.g., ref. ^121^), one-to-one interactions between an AI tutor and a student may displace other important learning sources: a peer’s alternative or even incorrect answer, for instance, can catalyse conceptual clarification, particularly in domains such as second-language acquisition^122–125^. More broadly, the sustained adoption of individualised AI tutors risks shifting pedagogical practices away from collaborative regulation, disagreement, and collective sense-making toward individualised competencies centred on prompting, filtering, and evaluating model outputs. GenAI tools are often framed and implemented with the explicit aim of playing a social role in learning, functioning as conversational partners, mentors, or collaborative agents. However, emerging evidence suggests that in practice their current design may reduce rather than support opportunities for collaboration, mentorship, and community building, with further implications for student motivation and sense of belonging^126,127^. These findings remain preliminary, and the limited number of studies addressing this issue empirically is itself telling, signalling that the social and relational dimensions of AI-mediated learning remain a largely overlooked area that warrants empirical investigation.

The fluent and seemingly authoritative outputs typical of GenAI further obscure the existence of alternative perspectives and compress the uncertainty that ordinarily accompanies reasoning and evaluation. Highly agreeable LLMs, while potentially helpful, might also risk weakening developmental learning by replacing dialogic friction with continuous affirmation and preference alignment^128–131^. In doing so, they may reduce learners’ encounters with resistant others whose perspectives cannot be fully controlled, anticipated, or personalised. By minimising ambiguity, these systems may habituate students to avoid effortful engagement, sustained problem-solving, and epistemic struggle^11^. When such habits develop early and are consistently reinforced across development—as often the case in school settings—they may foster patterns of passivity and overreliance on externally provided solutions^132^. Yet, learning sciences identify as key drivers of conceptual change precisely effort, struggle, and uncertainty resolution^133–135^: the same conditions that classrooms, particularly when informed by embodied and situated approaches, naturally afford through competing viewpoints, social negotiation, and the possibility of failure (e.g., refs. ^136–138^). Current AI design neither incorporates these conditions nor creates the structural affordances through which they might organically develop in classroom contexts: not because of permanent limitations, but because of the pedagogical assumptions shaping its implementation. These assumptions are not inevitable. Designed as a cognitive partner, engaging students through Socratic questioning, retrieval-practice, and reflective prompting rather than direct answer provision^139^, AI can foster deeper engagement and reflective thinking^140,141^), precisely by reintroducing the desirable difficulties that many implementations tend to eliminate. Yet, even these promising solutions - contingent on the kind of broader reflection on educational conditions that this article calls for - typically conceive the interaction as a one-to-one user-system relationship, leaving the collaborative and social dimensions of learning unaddressed. The human learner is positioned as a solitary interlocutor rather than as a participant in a broader social and epistemic community, a limitation that deliberate pedagogical design must equally confront.

If AI systems are deployed within, and designed to reproduce an already predominantly disembodied educational logic, the experiential conditions through which meaning is constructed risk being further marginalised: sustained engagement with multisensory materials, bodily action, and social partners (see refs. ^142,143^). The concern is not a generic loss of cognitive capacity, but the attenuation of the experiential conditions through which meaning is constructed^144^. Human concepts are rarely acquired from a single source of information. We do not learn what a cat is merely by hearing the word or viewing images, but by integrating visual, tactile, auditory, motor, and affective cues - seeing its movements, feeling its fur, hearing its purring, and interacting with it in context (for examples of multiple sensory channels in learning, see: e.g., ref. ^145^). Even when information is partial, learners construct robust concepts by coordinating signals coming from linguistic and non-linguistic sources^146^. When GenAI systems are employed in isolation, and their outputs are not complemented by other sensory, material, and social forms of engagement, learning risks being reduced to predominantly linguistic and screen-based exchanges. This narrowing of the channels through which understanding is formed is not new: it has long characterised traditional educational practices that rely predominantly on textbooks, written exercises, and abstract symbolic representations (e.g., ref. ^147^). Substituting pen-and-paper tests or textbooks with conversational interfaces does not, by itself, lead to a deeper understanding of abstract domains.

By contrast, inquiry-based and collaborative approaches grounded in a multi-technological mindset, combining low-tech materials with advanced digital tools, such as hands-on physical models alongside immersive simulations and game-based environments, are better positioned to support the integration of multiple informational channels through which robust knowledge develops (e.g., ref. ^147^). This is the vision of education as a multi-technological cognitive ecology, within which AI systems could play a meaningful role, if designed accordingly. Initiatives such as *Learn Your Way*^148^, which transforms traditional textbooks into multimedia learning materials including audio lessons, narrated slides, and mind maps tailored to learners’ preferences, point in this direction. It suggests the current misalignment as the consequence of the pedagogical logic within which AI systems have so far been designed and deployed, one that has yet to fully incorporate what learning sciences tell us about how human understanding is built.

### Goal gap

A second, closely related gap concerns the overlap between AI development and established educational environments: the *goal* gap. Where the pedagogical gap concerns how learning and cognition are conceptualised, the goal gap concerns the misalignment between the aims implicitly promoted by current technologies and the broader goals education is meant to serve. Also, this gap predates AI. Educational theory and policy emphasise the development of collaborative capacities, ethical formation, and critical thinking (ref. ^149^ also, e.g., refs. ^150,151^). Yet educational systems remain organised around inherited logics of efficiency, standardisation, and content coverage, making these goals difficult to operationalise (e.g., ref. ^152^). Technologies introduced without an explicit interrogation of educational aims do not challenge this misalignment: adapted to it, they reproduce and amplify it^153^. GenAI is no exception. Introduced within existing institutions, it aligns with the aims already implicit in those, narrowing educational discourse toward measurable outcomes (performance metrics, assessment efficiency, and standardised achievement) and obscuring the distinction between learning as a developmental process and performance as a measurable output^87^.

This becomes visible in recent educational initiatives claiming to compress traditional curricula to a few hours per day through extensive AI use (e.g., Alpha School). If academic instruction can indeed be radically compressed, this exposes a deeper set of questions that educational systems have so far avoided confronting. Whether the primary aim of schooling should solely be the optimisation of academic achievement; whether the time conventionally spent on curricula (8 h, or more, per day) has truly been worthwhile; if schooling might have always served broader functions that metrics fail to capture; and whether those metrics have themselves contributed to obscuring what education is, or should be, ultimately for. Once capacities are reduced to what can be easily articulated, assessed, and scaled, more fundamental educational functions risk being marginalised: socialisation (how we become part of particular social, cultural, and political identities) and individual agency (the process through which autonomy in thinking and acting develops)^2^. Higher-order capacities, including metacognitive engagement, epistemic responsibility, and the tolerance of uncertainty, risk being rendered secondary, subjected to performance-oriented metrics that reinforce a view of learning in terms of short-term outputs rather than developmental trajectories^154^.

This reductive tendency is further reflected in the strongly STEM-centric orientation of current AI research in education. Important as STEMs are, implementations focus predominantly on computational, mathematical, and programming skills^155^ at the expense of interpretive understanding, ethical reasoning, social participation, and creativity^156,157^. Yet these capacities are not separable from STEM competencies: they are the conditions under which scientific and technological knowledge can be reflexively and responsibly applied. A genuinely 21st-century education should resist this fragmentation, cultivating instead intellectually flexible, creative, and socially attuned individuals equipped with the reflective and interpersonal capacities needed to navigate increasingly complex and uncertain worlds^158^. In this sense, we should see the introduction of GenAI not as a source of instructional efficiency alone, but as a force that compels us to reconsider the purposes, values, and broader functions of education itself.

### Confabulations

A final concern relates to “hallucinations” or “confabulations” in GenAI systems, whereby incorrect or fabricated information (text or images) is presented fluently and with unwarranted confidence^159–161^. Despite mitigation efforts^162^, AI models continue to generate plausible but unfounded claims^163^, requiring external verification to establish reliability^164^, given potentially harmful consequences^165,166^.

In educational settings, this property carries both risks and a potential advantage^167^. Learners who, by definition, lack the background knowledge required for independent verification may struggle to distinguish accurate from spurious content, both delivered with equal authority^168^. Sustained reliance on such systems may further encourage the outsourcing of judgment and synthesis to the model, particularly among students with fewer supports, normalising passive knowledge consumption rather than the critical scrutiny, evidential reasoning, and civic values that education seeks to cultivate^169^. The advantage, however, is that awareness of hallucination can itself become a pedagogical resource, making verification, source-checking, and—importantly - collaborative fact evaluation integral rather than optional components of AI-supported learning^170^.

This structural tendency carries at least three educational implications. First, curricula may need to explicitly incorporate verification and source evaluation not as isolated competencies but as practices embedded across subjects, sensory channels, and learning modalities, drawing on diverse tools and pedagogies rather than treating them as add-ons to existing instruction. Second, GenAI systems themselves may require redesign so that, beyond usability and accessibility, they actively scaffold epistemic agency, critical reasoning, and collaborative inquiry across modalities and social configurations, rather than positioning the learner as a solitary recipient of authoritative outputs. Third, and more fundamentally, the persistence of confabulation tendencies calls into question the institutional role of schools as epistemic authorities.

Historically, knowledge within formal education has been stabilised through teachers, textbooks, and curricula, which defined what counted as *relevant* and *reliable* information within a given sociocultural context. GenAI disrupts this arrangement by introducing an additional, opaque, and probabilistic source of content whose outputs do not map cleanly onto established standards of authority. As a result, educational institutions can no longer assume that information provided through technological systems will be trustworthy or appropriately bounded, where schools once held authority not only over what is accurate, but over what is worth knowing and when, and must instead reconfigure how epistemic trust, responsibility, and accountability are distributed across teachers, learners, and tools. As students increasingly encounter information that appears authoritative yet may be inaccurate, independent verification (e.g., cross-checking claims across multiple sources, models, and media) should become a core component of learning, cultivating habits of evidence-based inquiry rather than merely mitigating error.

## Conclusions: multitechnological ecology

**Fig. 1:**
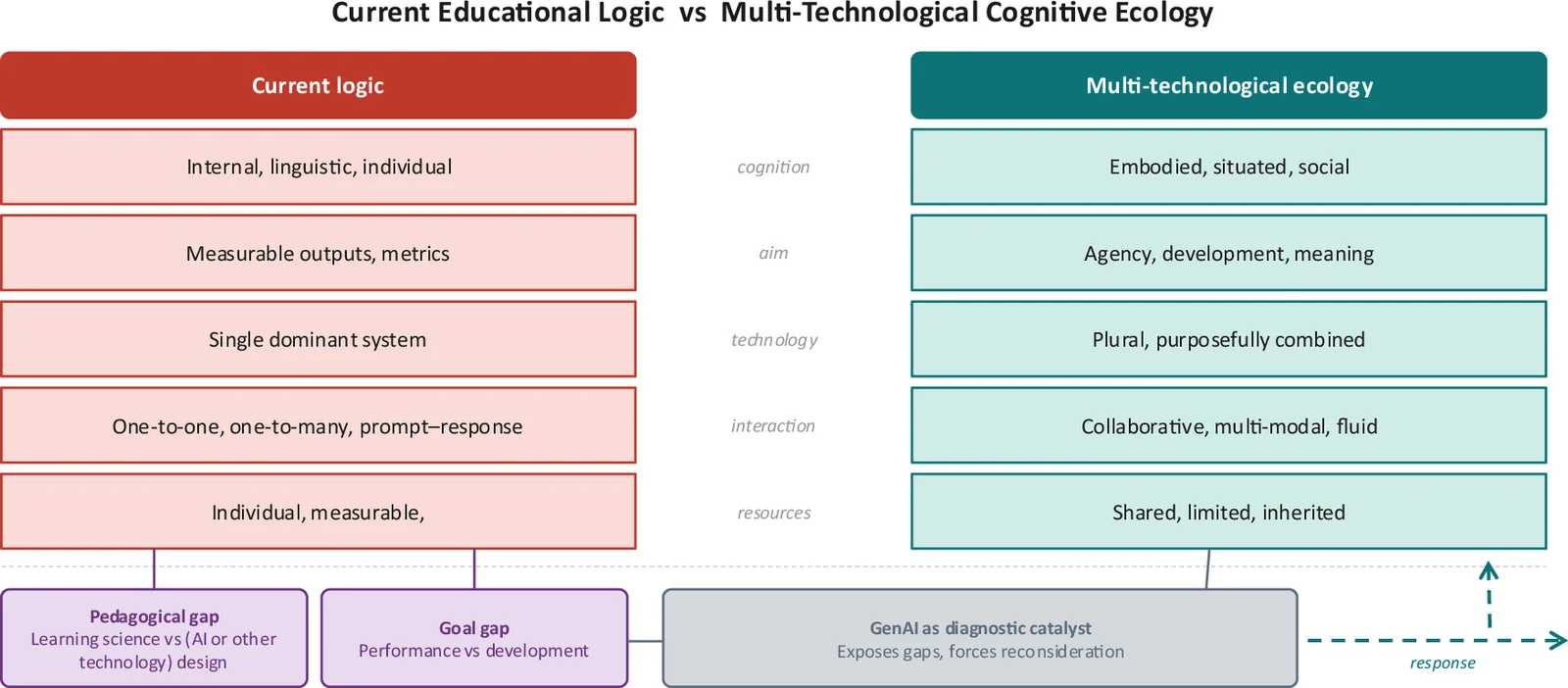
Current Educational Logic vs Multitechnological Cognitive Ecology.

These considerations cannot be addressed by optimising individual tools or incrementally adjusting curricula. They require a broader reconfiguration. GenAI, precisely because it unsettles established assumptions about knowledge, authority, and learning, represents a catalytic opportunity to undertake it (for a summary, see Fig.1).

This opportunity has precedent. The COVID-19 pandemic exposed the fragility of inherited educational arrangements with unprecedented clarity^31,33^. Yet, the prevailing response was restorative rather than transformative, focused on recovering what had been lost rather than reconsidering what might be built in its place^171^. Crises alone do not produce transformations, which require a prepared conceptual and practical vocabulary. The vocabulary for such a reconfiguration already exists. From Dewey’s *learning by doing*^172^ to Papert’s constructionist vision of coding as epistemic practice^173^, from place-based education^174^ to ‘Learning by Design’^175,176^, from Bers’ framing of coding as a playground for young learners^177^ to contemporary research on embodied learning environments^178^, and more powerfully, to Montessori’s *prepared environment* (e.g., refs. ^93,179–182^, a rich lineage of educational theory and practice has long insisted that learning is most powerful when it is embodied, situated, collaborative, and materially grounded. What has been lacking is not the vision, but the institutional will, the technological ecology, and the socio-economic resources adequate to realise it.

Realising it requires conceiving of education as a *multi-technological cognitive ecology*: a deliberately designed configuration of diverse material, social, and digital resources that jointly scaffold learning in ways consistent with what cognitive science tells us about human understanding^99^. Within such an ecology, no single technology dominates: low-tech, embodied, and collaborative tools are combined with digital and AI-based resources according to pedagogical aims rather than market logic. This framing also carries an eco-sustainable dimension that cannot be ignored. AI systems are among the most resource-intensive technologies ever deployed in educational contexts, with substantial energy, water, and infrastructural costs^183^. Introducing them into schools without accounting for these costs risks reproducing, at a larger scale, the same logic of resource exploitation that education is increasingly called upon to counter. A multi-technological ecological approach implies instead a principle of deliberate use: combining tools according to their pedagogical affordances, sharing resources across learners and generations, and treating AI as one component among many rather than as a default infrastructure: using, not abusing, the technological resources available^184,185^.

Seen in this light, GenAI represents less a ready-made solution than a diagnostic catalyst. It exposes longstanding tensions between educational systems organised around efficiency and performance and the broader developmental aims education is meant to serve: agency, critical judgment, collaboration, and meaningful participation in shared epistemic practices. These tensions are not new: AI makes them impossible to ignore. Addressing them requires shifting the central question from how to integrate AI into existing schools to how schooling itself should be reimagined in light of what we know about cognition, learning, and the ecological conditions that sustain both. The answer, we suggest, lies not in the adoption of any single technology, but in the deliberate design of plural technological ecologies, combining embodied, social, material, and digital resources, that only together are capable of supporting the full range of human learning and development. AI is neither saviour nor threat. It is a stress test for education, exposing the limits of existing models and compelling a reconsideration of their aims, foundations, and ecological responsibilities. It is one tool among many. Above all, it is a catalyst for reimagining the technological arrangements that structure every day at school.

## Supplementary information

Transparent Peer Review file

## Data Availability

No datasets were generated or analysed during the current study.
