# Supplementary material for: Educating minds with generative AI
Source: Commun Psychol. 2026 Sep 14;4:128. doi: 10.1038/s44271-026-00522-8 (PMC13575178; doi:10.1038/s44271-026-00522-8)
Supplement: Supplementary file 1 [file 44271_2026_522_MOESM1_ESM.pdf]

nature portfolio

Peer Review File

Educating Minds with Generative AI

Corresponding Author: Dr Laura Desiree Di Paolo

This file contains all reviewer reports in order by version, followed by all author rebuttals in order by version.

Version 0:

Reviewer comments:

Reviewer #1

(Remarks to the Author)
This manuscript addresses a timely and important topic, i.e., th role of generative AI in schooling, and it does so from an ambitious theoretical perspective grounded in cognitive science and cognition. The central claim is that GenAI should not be seen only as another educational tool, but as an epistemic infrastructure that can reorganize the whole cognitive ecology of schooling, with two major consequences the authors call the pedagogical gap and the goal gap. This is clearly stated in the paper and it is potentially interesting for a broad readership, especially because the article tries to move beyond the usual discussion of risks and opportunities.

At the same time, in my opinion the manuscript in its current form remains too programmatic and not yet sufficiently convincing for publication in this journal. The main problem is that the article makes very broad and strong claims, but the evidential basis is often not strong enough for the level of generality of these claims. Many arguments are plausible and intellectually stimulating, but the paper often moves quickly from conceptual framing to normative conclusion without enough careful qualification. For example, the manuscript repeatedly suggests that GenAI structurally redistributes epistemic labour, consolidates pedagogical functions, risks eroding epistemic agency, and may habituate learners toward passivity and overreliance, but these claims are mostly argued at a conceptual level and only selectively supported by empirical evidence.

In it current form, the manuscipt lacks to provide a clear jusstification for its novelty. The vocabulary of epistemic infrastructure, redistribution of epistemic labour, and cognitive ecology gives the manuscript a distinctive framing, but the underlying concerns themselves are not entirely new. The paper acknowledges existing debates on AI in education, embodied learning, and the mismatch between educational technology and learning sciences, but it does not clearly show what is genuinely new beyond synthesizing these strands under a broader philosophical umbrella. In its current version, the manuscript risks reading more like a perspective essay that redescribes known concerns in a richer conceptual language than a paper that advances a clearly delimited new argument.

I also found the treatment of prior educational technologies to be too simplistic. The paper contrasts earlier tools as largely passive, specialised, and temporally bounded, whereas GenAI is presented as active, persistent, and generalist. This contrast is rhetorically effective, but it feels historically overstated. Many earlier educational technologies also shaped cognition, redistributed authority, structured interaction, and influenced pedagogical norms in non-neutral ways. By drawing the distinction too sharply, the manuscript creates an artificial discontinuity that weakens the credibility of the argument.

Related to this, the manuscript often relies on binary oppositions that are too clean: embodied versus disembodied, collaborative versus individualised, developmental aims versus performance metrics, friction-rich classrooms versus frictionless AI systems. These distinctions are useful heuristically, but in practice educational settings are much messier. There are embodied and collaborative uses of digital tools, there are highly passive classroom practices without AI, and there are cases where carefully designed AI systems may support reflection rather than reduce it. Because the paper is written in a very assertive style, these nuances are not visible enough.

Another important issue is the manuscript’s engagement with empirical literature. The paper cites many sources, including recent papers, but the discussion remains selective and sometimes one-sided. There is much emphasis on risks such as cognitive offloading, deskilling, hallucinations, and overreliance, while positive or mixed evidence is treated more briefly. This creates the impression that the conclusion is decided in advance. Since the article wants to intervene in a psychologically and educationally relevant debate, it should engage more systematically with counter-evidence and with situations in which GenAI may indeed scaffold learning, especially when teacher guidance, collaborative design, or domain

constraints are present. At present, the manuscript feels more like an argument for caution than a balanced scholarly assessment.

The section on the “goal gap” raises an important point, but here too the argument remains broad and underdeveloped. The manuscript claims that AI tends to reinforce efficiency, standardisation, and measurable performance, at the expense of agency, critical judgment, and socialisation.

The manuscript is generally well written, but the prose is sometimes too dense, repetitive, and rhetorically loaded. Several paragraphs rely on long sentences and accumulations of abstract nouns, which makes the central message harder to follow than necessary. In addition, some formulations sound stronger than the available evidence justifies. In addition, on reproducibility, this is a conceptual and argumentative paper rather than an empirical study, so the usual standards of reproducibility do not apply in the same way.

Although this manuscript has potential as a provocative and thought-provoking perspective, in its current form it is not sufficiently precise, balanced, or empirically grounded, especially given the journal aimed for publication.

Reviewer #3

(Remarks to the Author)
The article proposes an ambitious theoretical reflection on the educational significance of generative artificial intelligence. Its main merit lies in shifting the focus from the instrumental discussion of AI's "uses" in the classroom to a structural interpretation: generative AI is not simply another tool, but a technology that reconfigures the cognitive, pedagogical, and epistemic ecologies of schooling. It is an intellectually stimulating work, well-written and supported by extensive and very recent literature. It provides a valuable and novel framework for interpreting the field of education and AI, although some of its theses would require greater pedagogical specificity and more robust empirical support to be fully convincing.

The article's originality lies not so much in discovering that AI poses educational risks or challenges—something already widely discussed—as in the interpretive framework from which these challenges are addressed. The shift from an instrumental view of AI to its understanding as an epistemic infrastructure and as a reorganizer of cognitive ecologies is the manuscript's most distinctive contribution. This way of framing the problem sets it apart from works more focused on performance, personalization, efficiency, or applied ethics.
Also original is the attempt to articulate the current debate on educational AI with the tradition of 4E cognition and with an ecological view of schooling. This approach enriches a field where technocentric or excessively functionalist perspectives often predominate.
Overall, I consider the article to be a work of high intellectual quality, relevant and timely. Its main contribution is to offer a powerful theoretical framework for understanding generative AI as a reconfigurator of school ecology, and not just as a support tool. This contribution is indeed novel and will be of interest to other members of the academic community, especially those who investigate the relationship between technology, cognition, and pedagogy. The text is compelling in its overall orientation and critical capacity, although some conclusions may need stronger empirical support and a more nuanced formulation. Its most likely influence will be not so much to close debates as to open new questions and provide useful categories for rethinking the field of how to teach and learn with AI from a holistic perspective.

One possible criticism is that the article diagnoses the risks of generative AI very well, but develops pedagogical alternatives less effectively. It states that education must be redesigned as a pluralistic, embodied, collaborative, and critical multi-technology ecology, but this proposal remains vaguely formulated.
Therefore, I suggest adding a more proactive final section that specifies design principles and pedagogical action for integrating AI into educational contexts or environments.
With these improvements, the manuscript can become a valuable contribution that can be cited within the contemporary academic debate on AI, learning, and education.

In conclusion, I congratulate the authors on their well-crafted article. It is dense, complex, and thought-provoking. Reading it has given me an original perspective on the problem of the impact of AI on schooling.

Version 1:

Reviewer comments:

Reviewer #3

(Remarks to the Author)
Dear Editor, I believe the authors of the article have adequately addressed the reviewers' comments and revised the text. Therefore, I support the publication of this article in its new version.
Thank you

Open Access This Peer Review File is licensed under a Creative Commons Attribution 4.0 International License, which permits use, sharing, adaptation, distribution and reproduction in any medium or format, as long as you give appropriate credit to the original author(s) and the source, provide a link to the Creative Commons license, and indicate if changes were made.
In cases where reviewers are anonymous, credit should be given to 'Anonymous Referee' and the source.
The images or other third party material in this Peer Review File are included in the article's Creative Commons license, unless indicated otherwise in a credit line to the material. If material is not included in the article's Creative Commons license and your intended use is not permitted by statutory regulation or exceeds the permitted use, you will need to obtain permission directly from the copyright holder.
To view a copy of this license, visit https://creativecommons.org/licenses/by/4.0/

Reviewers' comments: *black italic*
Direct response to reviewer: black (indented)
In text modifications: blue (indented)

Dear Editor, dear Reviewers,

We extend our sincere gratitude for your valuable and comprehensive feedback. We have addressed the reviewers' comments by directly responding to them in this document (black indented text) or through in-text modifications. Their insightful comments prompted us to engage in new debates, which are reflected in our direct responses. We have incorporated these debates as much as possible into our in-text modifications. References for the in-text modifications are provided at the end of the revised manuscript.

EDITOR'S COMMENTS:

Your core contribution needs to be fleshed out to establish as a framework of practical relevance for further research and practical implications of the use of GenAI. Please include a forward-looking section where, based on your previous criticism/diagnosis of the risk of GenAI, the design principles and pedagogical action for integrating AI into educational contexts are specified. We would expect this to span two to three paragraphs, and ideally, be accompanied by an illustration or schema.

Dear Editor,

We are deeply grateful for the time and care dedicated to reviewing our manuscript, and for the constructive guidance provided throughout this process. We have revised the manuscript thoroughly in response to all flagged comments, addressing each as fully as possible within the text itself. One editorial suggestion warrants an explicit response here: the proposal to add a dedicated final section outlining forward-looking recommendations. After careful consideration, we concluded that a standalone section would not adequately serve this purpose. Instead, we have restructured and reoriented the manuscript as a whole to embed a more balanced and forward-looking perspective throughout, which we believe produces a more coherent and integrated argument. We wish to be explicit on one point that may not have been sufficiently clear in the original submission: we are not opposed to AI implementation in educational settings - nor, in principle, to any technology. On the contrary, one of us is actively engaged in the redesign of AI tools in precisely the direction this paper advocates. Our aim was never to issue a verdict on AI, but to provide a principled

framework for thinking about the conditions under which any technology, AI especially, can genuinely support learning.

In response to the appreciative comments of Reviewer 2, we have revised and expanded the concluding section of the manuscript to articulate a clearer pathway toward design principles and pedagogical action. We have nonetheless chosen to leave this pathway deliberately open rather than prescriptive, for reasons we elaborate in our response to Reviewer 2 below. In brief: a genuine reconfiguration of educational systems is inseparable from its material, institutional, and spatial conditions. These conditions vary enormously across contexts and cannot be specified in advance. This paper therefore aims to establish the principles from which more context-specific implementation frameworks might be developed in future work. We hope these revisions address the Editor's concerns, and we remain available for any further clarification.

We have also implemented notes within the manuscript text. Accordingly, for instance:

P.7: Ongoing post-deployment modifications, including fine-tuning, alignment adjustments, and policy-driven constraints, can substantially alter model behaviour over time, determining which kinds of content are generated, emphasised, or suppressed, and reflecting shifting normative and institutional priorities [83]. Recent public debates surrounding the post-release modification of commercial language models (e.g., alignment changes in the model *Grok*) illustrates how such systems can be continuously adjusted to reflect evolving organisational or political commitments, underscoring that GenAI systems do not merely reproduce biases inherited from training data, but actively incorporate value judgements introduced during deployment and governance.

P.12: This becomes visible in recent educational initiatives claiming to compress traditional curricula to a few hours per day through extensive AI use (e.g., Alpha School). If academic instruction can indeed be radically compressed, this exposes a deeper set of questions that educational systems have so far avoided confronting. Whether the primary aim of schooling should solely be the optimisation of academic achievement; whether the time conventionally spent on curricula (8 hours, or more, per day) has truly been worthwhile; if schooling might have always served broader functions that metrics fail to capture; and whether those metrics have themselves contributed to obscuring what education is, or should be, ultimately for. Once capacities are reduced to what can be easily articulated, assessed, and scaled, more fundamental educational functions risk being marginalised: socialisation (how we become part of particular social, cultural, and political identities) and individual agency (the process through which autonomy in thinking and acting develops) [2]. Higher-order capacities, including metacognitive engagement, epistemic responsibility, and the tolerance of uncertainty, risk being rendered secondary,

subjected to performance-oriented metrics that reinforce a view of learning in terms of short-term outputs rather than developmental trajectories [154].

Finally, we provide a schema as requested. Since the Editor thinks it is necessary, we have gladly added to the paper. However, we are not sure how much our theoretical paper might improve due to its presence.

See below for more in depth in-text modifications.

REVIEWER #1

This manuscript addresses a timely and important topic, i.e., the role of generative AI in schooling, and it does so from an ambitious theoretical perspective grounded in cognitive science and cognition. The central claim is that GenAI should not be seen only as another educational tool, but as an epistemic infrastructure that can reorganize the whole cognitive ecology of schooling, with two major consequences the authors call the pedagogical gap and the goal gap. This is clearly stated in the paper and it is potentially interesting for a broad readership, especially because the article tries to move beyond the usual discussion of risks and opportunities.
At the same time, in my opinion the manuscript in its current form remains too programmatic and not yet sufficiently convincing for publication in this journal. The main problem is that the article makes very broad and strong claims, but the evidential basis is often not strong enough for the level of generality of these claims. Many arguments are plausible and intellectually stimulating, but the paper often moves quickly from conceptual framing to normative conclusion without enough careful qualification. For example, the manuscript repeatedly suggests that GenAI structurally redistributes epistemic labour, consolidates pedagogical functions, risks eroding epistemic agency, and may habituate learners toward passivity and overreliance, but these claims are mostly argued at a conceptual level and only selectively supported by empirical evidence.
In it current form, the manuscipt lacks to provide a clear jusstification for its novelty. The vocabulary of epistemic infrastructure, redistribution of epistemic labour, and cognitive ecology gives the manuscript a distinctive framing, but the underlying concerns themselves are not entirely new. The paper acknowledges existing debates on AI in education, embodied learning, and the mismatch between educational technology and learning sciences, but it does not clearly show what is genuinely new beyond synthesizing these strands under a broader philosophical umbrella. In its current version, the manuscript risks reading more like a perspective essay that redescribes known concerns in a richer conceptual language than a paper that advances a clearly delimited new argument.

Dear Reviewer, we extend our sincere gratitude for the time you invested in reviewing our manuscript and for the valuable suggestions you provided. We hope this revised version more effectively grounds our work in current empirical and theoretical research

and shows its novelty. We have revised the empirical supporting material throughout the manuscript, trying to address any concerns you might have.

For instance, accordingly, we add:

P.5: This shift is best understood by conceptualising GenAI as an epistemic infrastructure and as a transformative element within existing ones [51]. Epistemic infrastructures, whether physical or institutional (e.g., libraries, laboratories, hospitals, or ritual practices), do more than provide access to knowledge: they organise how knowledge is produced, circulated, evaluated, and legitimised. By structuring epistemic activities and regulating epistemic effort, they stabilise the norms that define what counts as competent participation in cultural practices [52,53]. When introduced and institutionalised into education, GenAI systems might effortlessly assume this infrastructural role. AI-powered teaching management systems (e.g., Toddle, ManageBac) deliberately consolidate core functions (curriculum planning, instructional support, learner assessment, and progress reporting) within a unified, algorithmically mediated environment, imposing a mandatory participation structure on all educational actors, including teachers, administrators, secretaries, principals, and parents, who must adapt their practices to the system's operational logic regardless of individual preference or unaligned with prior workflow. Modifying standards of explanation and assessment, regulating teaching and learning activities, they reshape what counts as legitimate participation in the educational system itself [54] (see also:[55]).

Pp.10-11: More broadly, the sustained adoption of individualised AI tutors risks shifting pedagogical practices away from collaborative regulation, disagreement, and collective sense-making toward individualised competencies centred on prompting, filtering, and evaluating model outputs. GenAI tools are often framed and implemented with the explicit aim of playing a social role in learning, functioning as conversational partners, mentors, or collaborative agents. However, emerging evidence suggests that in practice their current design may reduce rather than support opportunities for collaboration, mentorship, and community building, with further implications for student motivation and sense of belonging [126,127]. These findings remain preliminary, and the limited number of studies addressing this issue empirically is itself telling, signalling that the social and relational dimensions of AI-mediated learning remain a largely overlooked area that warrants empirical investigation.
The fluent and seemingly authoritative outputs typical of GenAI further obscure the existence of alternative perspectives and compress the uncertainty that ordinarily accompanies reasoning and evaluation. Highly agreeable LLMs, while potentially helpful, might also risk weakening developmental learning by replacing dialogic friction with continuous affirmation and preference alignment [128–131]. In doing so, they may reduce

learners' encounters with resistant others whose perspectives cannot be fully controlled, anticipated, or personalised. By minimising ambiguity, these systems may habituate students to avoid effortful engagement, sustained problem-solving, and epistemic struggle [11]. When such habits develop early and are consistently reinforced across development - as often the case in school settings - they may foster patterns of passivity and overreliance on externally provided solutions [132]. Yet, learning sciences identify as key drivers of conceptual change precisely effort, struggle, and uncertainty resolution [133–135]: the same conditions that classrooms, particularly when informed by embodied and situated approaches, naturally afford through competing viewpoints, social negotiation, and the possibility of failure (e.g., [136–138]). Current AI design neither incorporates these conditions nor creates the structural affordances through which they might organically develop in classroom contexts: not because of permanent limitations, but because of the pedagogical assumptions shaping its implementation. These assumptions are not inevitable. Designed as a cognitive partner, engaging students through Socratic questioning, retrieval-practice, and reflective prompting rather than direct answer provision [139], AI can foster deeper engagement and reflective thinking [140,141]), precisely by reintroducing the desirable difficulties that many implementations tend to eliminate. Yet, even these promising solutions - contingent on the kind of broader reflection on educational conditions that this article calls for - typically conceive the interaction as a one-to-one user-system relationship, leaving the collaborative and social dimensions of learning unaddressed. The human learner is positioned as a solitary interlocutor rather than as a participant in a broader social and epistemic community, a limitation that deliberate pedagogical design must equally confront.

P.13: In educational settings, this property carries both risks and a potential advantage [167]. Learners who, by definition, lack the background knowledge required for independent verification may struggle to distinguish accurate from spurious content, both delivered with equal authority [168]. Sustained reliance on such systems may further encourage the outsourcing of judgment and synthesis to the model, particularly among students with fewer supports, normalising passive knowledge consumption rather than the critical scrutiny, evidential reasoning, and civic values that education seeks to cultivate [169]. The advantage, however, is that awareness of hallucination can itself become a pedagogical resource, making verification, source-checking, and - importantly - collaborative fact evaluation integral rather than optional components of AI-supported learning [170].

I also found the treatment of prior educational technologies to be too simplistic. The paper contrasts earlier tools as largely passive, specialised, and temporally bounded, whereas GenAI is presented as active, persistent, and generalist. This contrast is rhetorically effective, but it feels historically overstated. Many earlier educational technologies also shaped cognition,

redistributed authority, structured interaction, and influenced pedagogical norms in non-neutral ways. By drawing the distinction too sharply, the manuscript creates an artificial discontinuity that weakens the credibility of the argument.

We thank the reviewer for this comment and acknowledge that our previous formulation may have lacked clarity in some points, likely as a consequence of space constraints. We agree with the reviewer's point in principle. All materiality - from the mundane (desks, chairs, corridors, pencils) to the specialised (computers, interactive whiteboards, or Montessori sensory materials or the "Puzzle Maps") - shapes cognition in ways that are medium-specific. In fact, we mentioned already in the manuscript:

P.2: "Moreover, formal education has never operated independently of technology. From books, blackboards, and abacuses to calculators and computers; from individual desks to open-space classrooms and digital e-learning platforms, schooling has always relied on material artefacts to scaffold learning. These artefacts do not merely convey information; they become functionally integrated into cognitive activity, shaping how learners attend, reason, remember, and act. In this sense, the skills and forms of knowledge cultivated in schools are inseparable from the tools and technologies through which they are developed (for an account of hybrid cognition: [3]). Thus, even if not always explicitly acknowledged, schools have always been hybrid, multi-technological cognitive ecologies, in which tools, materials, norms, and routines jointly structure learners' agency and epistemic habits over development (see, e.g.: [4–6])."

To strengthen the point, we have accordingly also modified the text:

Pp.5-6: Most educational tools primarily mediate access to information or scaffold how content is presented. Whether books, overhead slides, or physical and digital games, they support human activity without acting on the user's behalf: their functions are typically specialised, temporally bounded, and fundamentally passive. A book, for example, is used intermittently, contains fixed and often partial information, and cannot adapt to changing contexts or tasks. It is worth noting, however, that there has always been some educational materials and environments deliberately designed not to support a single specialised function, but to enable multiple operations almost simultaneously. Comenius's *Orbis Pictus* (1658) was conceived to teach vocabulary, language, and world knowledge through the integrated use of image and text within a single volume [56]. Papert's Logo programming environment was designed to merge mathematical reasoning, problem-solving, and epistemic reflection into a unified practice, in which debugging code and developing conceptual understanding were not separate activities but facets of the same process [57–59]. Abbott's *Flatland* (1884), while not intentionally a pedagogical instrument, might operate as a lesson [60] and, simultaneously, inform about a historical period and elicit a

critical representation of it. Even purpose-built multisensory artefacts, such as the Montessori ‘Stamp Game’ for mathematics or ‘Puzzle Maps’ for geography, explicitly designed to be self-correcting and to engage multiple cognitive functions and sensory channels simultaneously, remain bounded in their affordances (se, e.g., [61–63]. A physical globe cannot autonomously provide greater detail about a particular country or city on demand; the *Orbis Pictus* cannot answer a question it does not contain; the Logo environment requires a teacher or peer to provide affective scaffolding; *Flatland* cannot adapt its geometry lesson to the reader's understanding, nor elaborate its social critique beyond what the text can afford. In each of these cases, multifunctionality was deliberate. Yet, each artefact carries a ceiling determined by its materiality and its author's intent, and the user must frequently move outside the object itself to seek elaboration, clarification, or assessment.

AI-based conversational systems operate on a qualitatively different register, functioning as active, or at least semi-active, participants in epistemic processes. Although they do not possess human-like agency, nor access to the sensory channels that some other educational devices engage, they can plan and execute sequences of actions on a user's behalf with limited supervision [64], enabling increasing degrees of functional autonomy. Continuously updated and reconfigured after deployment, they become enduring and highly adaptable components of educational environments rather than static tools. This combination of autonomy and adaptability allows GenAI to perform epistemic functions that, until recently, were largely reserved for human cognition or required constant oversight, including problem framing, explanation generation, evaluation, and the selection and organisation of relevant information, such as checking test responses against an answer key [65–67], as well as forms of mental and emotional support in educational contexts and beyond [68–74]. Within a single interface, they can subsume functions ranging from instruction and error correction to adaptive scaffolding and proto-emotional support such as encouragement, dynamically calibrating to the user's level of knowledge and requiring comparatively little prior competence to access [75]. Their adoption thus marks a qualitative shift: from tools that support human activity while rarely combining multiple properties simultaneously (e.g., [38,76]), to systems that increasingly participate in, and may autonomously reorganise the epistemic processes themselves. This is not a question of moral hierarchy between technological paradigms. It is, however, necessary to acknowledge that the convergence of multiple (pedagogical) functions within a single system substantially amplifies both the possibilities and the associated risks, a scaling effect that is qualitatively distinct in the case of AI technologies.

Restricting our analysis to school-implented artefacts [we substituted the reference to *prior* with *different, other, most …*], they are generally bounded by the vectors, directions, and proportions through which they mediate learning. A Montessori "prepared environment", for instance, affords rich opportunities for social interaction, collaboration,

and self-directed engagement, particularly when contrasted with more traditional classroom arrangements. Yet even this highly intentional environment must be considered holistically, embedded within its broader pedagogical framework, and necessarily distributed across times, places, and people to fulfil functions such as parental communication, task preparation, memory consolidation, and curriculum progression, given that Montessori methodology foregoes traditional grading and standardised assessment. By comparison, non-AI digital platforms such as *Seesaw* already enable the compounding of several such functions within a single environment, combining parental communication, task assignment, and curriculum tracking. AI-powered systems such as *Toddle* extend this capacity considerably further: in principle, a single system could simultaneously support curriculum planning, assignment scheduling, parental communication, and logistical coordination (e.g., scheduling field trips). Crucially, once such a plan is instantiated, students are also drawn into the same environment to manage their assignments and timetables. At the same time, the system may, in principle and - more or less - transparently, concurrently readjust schedules, track curriculum coverage, generate assessments, and communicate with families. We wish to be explicit that we attribute *no hierarchy,* moral or in terms of effectiveness, to any of these systems or to the cognitive reorganisations they produce: each entails both affordances and constraints, and indeed we wish for a *mixed* environment, in which old, different, and new coexist. **Nevertheless, a meaningful asymmetry warrants attention.** Access to a book presupposes the ability to read and interpret it, yet the book itself cannot respond beyond its content, nor extend its cognitive influence beyond its materiality: its typography, authorship, sensory qualities, or visual organisation. A reader seeking elaboration, clarification, or self-assessment must typically consider additional materials, consult another person, or choose a different medium. Even an exercise book with an answer key generally requires the learner to consult a separate section or supplementary sheet. AI-based conversational systems, by contrast, can subsume all of these functions within a single interactional interface, and may additionally provide “proto” or otherwise seemingly affective scaffolding, such as encouragement, when prompted. Although LLMs cannot, in principle, respond beyond their training data, the generative nature of their reasoning works in both directions: the same capacity that produces hallucinations and confabulation is also a source of creative potential. The creative reassemblage of existing content may in fact allow them to go beyond any single source. And their training data already vastly exceeds what any book or encyclopedia could contain.

Related to this, the manuscript often relies on binary oppositions that are too clean: embodied versus disembodied, collaborative versus individualised, developmental aims versus performance metrics, friction-rich classrooms versus frictionless AI systems. These distinctions are useful heuristically, but in practice educational settings are much messier. There are embodied and collaborative uses of digital tools, there are highly passive classroom practices without AI, and

there are cases where carefully designed AI systems may support reflection rather than reduce it. Because the paper is written in a very assertive style, these nuances are not visible enough.

Accordingly:

P.3: While some studies suggest that pedagogically-aligned implementations of AI can support learning (e.g., [14–16]), emerging evidence indicates that intensive and unguided use of LLM-based systems may erode precisely those (meta)cognitive capacities (e.g., critical thinking, epistemic ownership) that underpin effective learning and that education is expected to cultivate [17]. This raises significant questions about the impact of such technologies on students' cognitive development, even in cases where they are adopted with the explicit aim of enhancing it [18]. These findings, however, remain preliminary, and they are situated within a broader and as yet unresolved debate over whether AI systems primarily function as instruments of cognitive enhancement or, conversely, as drivers of cognitive deskilling (e.g., [19,20]). It is against this backdrop of empirical uncertainty that the absence of sustained reflection on pedagogical and institutional conditions of deployment becomes especially consequential [21–23] (see also: [20]).

We thank the reviewer for this observation, since we acknowledge that our previous framing may have inadvertently conveyed a critical stance toward AI adoption *per se*. We consider it an oversight worth addressing explicitly: we are not opposed to the adoption of AI, nor, in principle, to any technology. What matters, from our perspective, is not the technology itself but the *programmatic intentionality* with which it is implemented: the aims it is designed to serve, the pedagogical framework within which it is embedded, and the institutional conditions under which it is introduced. The central argument of our work was never that AI is inherently problematic, but rather that the consequences of any technology are critically dependent on the context and logic of its deployment. We have therefore made several revisions to better reflect the complexity and mixed nature of the existing evidence, ensuring that the range of ways in which AI has been implemented to promote learning is more adequately represented.

Accordingly, we add:

P.8: The *pedagogical* gap, situated within a broader disconnection between learning science and technology design [89], captures the misalignment between contemporary 4E-informed understandings of learning and the pedagogical assumptions implicitly encoded in current AI systems. This misalignment, however, should not be read as an indictment of AI as such. Current GenAI systems are largely designed and deployed within, and therefore reproduce, a pedagogical logic that operationalises learning in predominantly disembodied, abstract, and linguistic terms [34,90]. A logic that predates AI and remains pervasive across

mainstream educational practice. The friction with research emphasising learning as an active, situated, embodied, and socially mediated process (e.g., [42,44,91–100]) is therefore not an intrinsic property of AI systems, but rather a consequence of the assumptions baked into their design. To the extent that a pedagogical gap exists, it reflects the broader dominance of disembodied pedagogical frameworks in educational technology more generally, of which current AI implementations are one instantiation.
This becomes concrete when considering the contrast between 4E-inspired pedagogical approaches and the design of current AI systems. Where 4E-informed pedagogies promote learning practices grounded in movement, active participation, and collaboration (e.g., [101]), in which students engage in perceptual, sensory, and motor activities to deepen their understanding of ideas and concepts [102], current educational AI systems continue to be conceived and realised as individualised, screen- and keyboard-based assistants [35,87], structurally limiting opportunities for bodily engagement, social interaction, and interpersonal understanding.

P.9: It is worth noting that some implementations have begun to explore alternative pathways aimed at closing this gap, embedding GenAI within mixed reality environments to afford more active and cognitively engaging learning experiences [104,105], or integrating it within embodied, collaborative pedagogical frameworks to promote more inclusive outcomes, including for students with special educational needs [106]. While promising, however, these cases remain exceptions, and their capacity to produce significant or lasting change may be structurally constrained. In our view, the gap reflects a persistent tension between "progressive" accounts of learning and more "conservative" institutional and technological arrangements, inherited physical spaces, tools, and social structures, that continue to shape schooling practices and resist viewing educational institutions as multi-technological cognitive ecologies. When GenAI is introduced into environments governed by such arrangements, it risks stabilising rather than challenging outdated pedagogical assumptions, regardless of the intentions behind its design. Closing the gap, therefore, requires more than technical innovation: it demands broader pedagogical, institutional, and cognitive reflection on the conditions under which any technology is deployed, a point well captured by Weidlich and colleagues' reminder that "the efficacy of learning is determined by the instructional method, not the medium through which it is delivered" ([107]: p.2).

P.10: By contrast, GenAI systems increasingly compress multiple pedagogical functions into a seamless interface. Explanation, feedback, evaluation, and content generation are delivered instantaneously within the same interactional loop, typically through chat-based or voice-based exchanges, smoothing over the frictions and ambiguities that play a pivotal role in learning [119,120]. This design logic rests on assumptions that construe cognition as primarily internal, linguistic, and individual: not an intrinsic property of AI, but one that

paradoxically prevents it from realising its full educational potential. This becomes evident when considering what current implementations foreclose.

According to the reviewer's comments, we have also tweaked the paragraphs so as to make our argument less categorical and more precise throughout the whole manuscript.

Another important issue is the manuscript's engagement with empirical literature. The paper cites many sources, including recent papers, but the discussion remains selective and sometimes one-sided. There is much emphasis on risks such as cognitive offloading, deskilling, hallucinations, and overreliance, while positive or mixed evidence is treated more briefly. This creates the impression that the conclusion is decided in advance. Since the article wants to intervene in a psychologically and educationally relevant debate, it should engage more systematically with counter-evidence and with situations in which GenAI may indeed scaffold learning, especially when teacher guidance, collaborative design, or domain constraints are present. At present, the manuscript feels more like an argument for caution than a balanced scholarly assessment.

We thank the reviewer for the opportunity to strengthen the manuscript's engagement with the empirical evidence substantially. Beyond the responses already provided above, we have amended the manuscript accordingly, adding references throughout (not all enumerated here but present in the manuscript and added to all modified paragraphs). For an example:

P.3: While some studies suggest that pedagogically-aligned implementations of AI can support learning (e.g., [14–16])...

We also thank the reviewer for having given us the chance to better express our point. We agree with the reviewer and maintain that the problem lies not in AI per se, but in the implicit pedagogical assumptions guiding its current design and deployment. The manuscript has been revised accordingly, reshaping the sections to express this idea more clearly:

P.8: The *pedagogical* gap, situated within a broader disconnection between learning science and technology design [89], captures the misalignment between contemporary 4E-informed understandings of learning and the pedagogical assumptions implicitly encoded in current AI systems. This misalignment, however, should not be read as an indictment of AI as such. Current GenAI systems are largely designed and deployed within, and therefore reproduce, a pedagogical logic that operationalises learning in predominantly disembodied, abstract, and linguistic terms [34,90]. A logic that predates AI and remains pervasive across mainstream educational practice. The friction with research emphasising learning as an

active, situated, embodied, and socially mediated process (e.g., [42,44,91–100]) is therefore not an intrinsic property of AI systems, but rather a consequence of the assumptions baked into their design. To the extent that a pedagogical gap exists, it reflects the broader dominance of disembodied pedagogical frameworks in educational technology more generally, of which current AI implementations are one instantiation.
This becomes concrete when considering the contrast between 4E-inspired pedagogical approaches and the design of current AI systems. Where 4E-informed pedagogies promote learning practices grounded in movement, active participation, and collaboration (e.g., [101]), in which students engage in perceptual, sensory, and motor activities to deepen their understanding of ideas and concepts [102], current educational AI systems continue to be conceived and realised as individualised, screen- and keyboard-based assistants [35,87], structurally limiting opportunities for bodily engagement, social interaction, and interpersonal understanding. [...]

<u>P.9</u>: It is worth noting that some implementations have begun to explore alternative pathways aimed at closing this gap, embedding GenAI within mixed reality environments to afford more active and cognitively engaging learning experiences [104,105], or integrating it within embodied, collaborative pedagogical frameworks to promote more inclusive outcomes, including for students with special educational needs [106]. While promising, however, these cases remain exceptions, and their capacity to produce significant or lasting change may be structurally constrained. In our view, the gap reflects a persistent tension between "progressive" accounts of learning and more "conservative" institutional and technological arrangements, inherited physical spaces, tools, and social structures, that continue to shape schooling practices and resist viewing educational institutions as multi-technological cognitive ecologies. When GenAI is introduced into environments governed by such arrangements, it risks stabilising rather than challenging outdated pedagogical assumptions, regardless of the intentions behind its design. Closing the gap, therefore, requires more than technical innovation: it demands broader pedagogical, institutional, and cognitive reflection on the conditions under which any technology is deployed, a point well captured by Weidlich and colleagues' reminder that "the efficacy of learning is determined by the instructional method, not the medium through which it is delivered" ([107]: p.2).

<u>Pp.10-11: see above</u>

The section on the "goal gap" raises an important point, but here too the argument remains broad and underdeveloped. The manuscript claims that AI tends to reinforce efficiency, standardisation, and measurable performance, at the expense of agency, critical judgment, and socialisation.

We sincerely thank the reviewer for this comment. We have modified the section accordingly:

Pp.12-13: A second, closely related gap concerns the overlap between AI development and established educational environments: the *goal* gap. Where the pedagogical gap concerns how learning and cognition are conceptualised, the goal gap concerns the misalignment between the aims implicitly promoted by current technologies and the broader goals education is meant to serve. Also this gap predates AI. Educational theory and policy emphasise the development of collaborative capacities, ethical formation, and critical thinking ([149] also, e.g., [150,151]). Yet educational systems remain organised around inherited logics of efficiency, standardisation, and content coverage, making these goals difficult to operationalise (e.g., [152]). Technologies introduced without an explicit interrogation of educational aims do not challenge this misalignment: adapted to it, they reproduce and amplify it [153]. GenAI is no exception. Introduced within existing institutions, it aligns with the aims already implicit in those, narrowing educational discourse toward measurable outcomes (performance metrics, assessment efficiency, and standardised achievement) and obscuring the distinction between learning as a developmental process and performance as a measurable output [87].

This becomes visible in recent educational initiatives claiming to compress traditional curricula to a few hours per day through extensive AI use (e.g., Alpha School). If academic instruction can indeed be radically compressed, this exposes a deeper set of questions that educational systems have so far avoided confronting. Whether the primary aim of schooling should solely be the optimisation of academic achievement; whether the time conventionally spent on curricula (8 hours, or more, per day) has truly been worthwhile; if schooling might have always served broader functions that metrics fail to capture; and whether those metrics have themselves contributed to obscuring what education is, or should be, ultimately for. Once capacities are reduced to what can be easily articulated, assessed, and scaled, more fundamental educational functions risk being marginalised: socialisation (how we become part of particular social, cultural, and political identities) and individual agency (the process through which autonomy in thinking and acting develops) [2]. Higher-order capacities, including metacognitive engagement, epistemic responsibility, and the tolerance of uncertainty, risk being rendered secondary, subjected to performance-oriented metrics that reinforce a view of learning in terms of short-term outputs rather than developmental trajectories [154].

This reductive tendency is further reflected in the strongly STEM-centric orientation of current AI research in education. Important as STEMs are, implementations focus predominantly on computational, mathematical, and programming skills [155] at the expense of interpretive understanding, ethical reasoning, social participation, and creativity [156,157]. Yet these capacities are not separable from STEM competencies: they are the conditions under which scientific and technological knowledge can be reflexively and

responsibly applied. A genuinely 21st-century education should resist this fragmentation, cultivating instead intellectually flexible, creative, and socially attuned individuals equipped with the reflective and interpersonal capacities needed to navigate increasingly complex and uncertain worlds [158]. In this sense, we should see the introduction of GenAI not as a source of instructional efficiency alone, but as a force that compels us to reconsider the purposes, values, and broader functions of education itself.

The manuscript is generally well written, but the prose is sometimes too dense, repetitive, and rhetorically loaded. Several paragraphs rely on long sentences and accumulations of abstract nouns, which makes the central message harder to follow than necessary. In addition, some formulations sound stronger than the available evidence justifies. In addition, on reproducibility, this is a conceptual and argumentative paper rather than an empirical study, so the usual standards of reproducibility do not apply in the same way.

We have amended the manuscript accordingly, wherever possible.

REVIEWER #2

The article proposes an ambitious theoretical reflection on the educational significance of generative artificial intelligence. Its main merit lies in shifting the focus from the instrumental discussion of AI's "uses" in the classroom to a structural interpretation: generative AI is not simply another tool, but a technology that reconfigures the cognitive, pedagogical, and epistemic ecologies of schooling. It is an intellectually stimulating work, well-written and supported by extensive and very recent literature. It provides a valuable and novel framework for interpreting the field of education and AI, although some of its theses would require greater pedagogical specificity and more robust empirical support to be fully convincing.

The article's originality lies not so much in discovering that AI poses educational risks or challenges—something already widely discussed—as in the interpretive framework from which these challenges are addressed. The shift from an instrumental view of AI to its understanding as an epistemic infrastructure and as a reorganizer of cognitive ecologies is the manuscript's most distinctive contribution. This way of framing the problem sets it apart from works more focused on performance, personalization, efficiency, or applied ethics.
Also original is the attempt to articulate the current debate on educational AI with the tradition of 4E cognition and with an ecological view of schooling. This approach enriches a field where technocentric or excessively functionalist perspectives often predominate.
Overall, I consider the article to be a work of high intellectual quality, relevant and timely. Its main contribution is to offer a powerful theoretical framework for understanding generative AI as a reconfigurator of school ecology, and not just as a support tool. This contribution is indeed novel and will be of interest to other members of the academic community, especially those who

investigate the relationship between technology, cognition, and pedagogy. The text is compelling in its overall orientation and critical capacity, although some conclusions may need stronger empirical support and a more nuanced formulation. Its most likely influence will be not so much to close debates as to open new questions and provide useful categories for rethinking the field of how to teach and learn with AI from a holistic perspective.

One possible criticism is that the article diagnoses the risks of generative AI very well, but develops pedagogical alternatives less effectively. It states that education must be redesigned as a pluralistic, embodied, collaborative, and critical multi-technology ecology, but this proposal remains vaguely formulated.
Therefore, I suggest adding a more proactive final section that specifies design principles and pedagogical action for integrating AI into educational contexts or environments.
With these improvements, the manuscript can become a valuable contribution that can be cited within the contemporary academic debate on AI, learning, and education.

We thank the reviewer for the time dedicated to our manuscript and for the positive feedback. We have amended the manuscript according to reviewer 1 comment. This has also allowed us to make our *re-design* position stronger throughout the manuscript, not only in the final section. We have also, though, amended the final section. Accordingly:

Pp.11-12: By contrast, inquiry-based and collaborative approaches grounded in a multi-technological mindset, combining low-tech materials with advanced digital tools, such as hands-on physical models alongside immersive simulations and game-based environments, are better positioned to support the integration of multiple informational channels through which robust knowledge develops (e.g., [147]). This is the vision of education as a multi-technological cognitive ecology, within which AI systems could play a meaningful role, if designed accordingly. Initiatives such as *Learn Your Way* [148], which transforms traditional textbooks into multimedia learning materials including audio lessons, narrated slides, and mind maps tailored to learners' preferences, point in this direction. It suggests the current misalignment as the consequence of the pedagogical logic within which AI systems have so far been designed and deployed, one that has yet to fully incorporate what learning sciences tell us about how human understanding is built.

P.13: In educational settings, this property carries both risks and a potential advantage [167]. Learners who, by definition, lack the background knowledge required for independent verification may struggle to distinguish accurate from spurious content, both delivered with equal authority [168]. Sustained reliance on such systems may further encourage the outsourcing of judgment and synthesis to the model, particularly among students with fewer supports, normalising passive knowledge consumption rather than the critical scrutiny, evidential reasoning, and civic values that education seeks to cultivate

[169]. The advantage, however, is that awareness of hallucination can itself become a pedagogical resource, making verification, source-checking, and - importantly - collaborative fact evaluation integral rather than optional components of AI-supported learning [170].

This structural tendency carries at least three educational implications. First, curricula may need to explicitly incorporate verification and source evaluation not as isolated competencies but as practices embedded across subjects, sensory channels, and learning modalities, drawing on diverse tools and pedagogies rather than treating them as add-ons to existing instruction. Second, GenAI systems themselves may require redesign so that, beyond usability and accessibility, they actively scaffold epistemic agency, critical reasoning, and collaborative inquiry across modalities and social configurations, rather than positioning the learner as a solitary recipient of authoritative outputs.

Pp. 14-15: These considerations cannot be addressed by optimising individual tools or incrementally adjusting curricula. They require a broader reconfiguration. GenAI, precisely because it unsettles established assumptions about knowledge, authority, and learning, represents a catalytic opportunity to undertake it.

This opportunity has precedent. The COVID-19 pandemic exposed the fragility of inherited educational arrangements with unprecedented clarity [31,33]. Yet, the prevailing response was restorative rather than transformative, focused on recovering what had been lost rather than reconsidering what might be built in its place [171]. Crises alone do not produce transformations, which require a prepared conceptual and practical vocabulary.

The vocabulary for such a reconfiguration already exists. From Dewey's *learning by doing* [172] to Papert's constructionist vision of coding as epistemic practice [173], from place-based education [174] to 'Learning by Design' [175,176], from Bers's framing of coding as a playground for young learners [177] to contemporary research on embodied learning environments [178], and more powerfully, to Montessori's *prepared environment* (e.g., [93,179–182], a rich lineage of educational theory and practice has long insisted that learning is most powerful when it is embodied, situated, collaborative, and materially grounded. What has been lacking is not the vision, but the institutional will, the technological ecology, and the socio-economic resources adequate to realise it.

Realising it requires conceiving of education as a *multi-technological cognitive ecology*: a deliberately designed configuration of diverse material, social, and digital resources that jointly scaffold learning in ways consistent with what cognitive science tells us about human understanding [99]. Within such an ecology, no single technology dominates: low-tech, embodied, and collaborative tools are combined with digital and AI-based resources according to pedagogical aims rather than market logic. This framing also carries an eco-sustainable dimension that cannot be ignored. AI systems are among the most resource-intensive technologies ever deployed in educational contexts, with substantial energy, water, and infrastructural costs [183]. Introducing them into schools without accounting

for these costs risks reproducing, at a larger scale, the same logic of resource exploitation that education is increasingly called upon to counter. A multi-technological ecological approach implies instead a principle of deliberate use: combining tools according to their pedagogical affordances, sharing resources across learners and generations, and treating AI as one component among many rather than as a default infrastructure: using, not abusing, the technological resources available [184,185].
Seen in this light, GenAI represents less a ready-made solution than a diagnostic catalyst. It exposes longstanding tensions between educational systems organised around efficiency and performance and the broader developmental aims education is meant to serve: agency, critical judgment, collaboration, and meaningful participation in shared epistemic practices. These tensions are not new: AI makes them impossible to ignore. Addressing them requires shifting the central question from how to integrate AI into existing schools to how schooling itself should be reimagined in light of what we know about cognition, learning, and the ecological conditions that sustain both. The answer, we suggest, lies not in the adoption of any single technology, but in the deliberate design of plural technological ecologies, combining embodied, social, material, and digital resources, that only all together are capable of supporting the full range of human learning and development. AI is neither saviour nor threat. It is a stress test for education, exposing the limits of existing models and compelling a reconsideration of their aims, foundations, and ecological responsibilities. It is one tool among many. Above all, it is a catalyst for reimagining the technological arrangements that structure every day at school.

We appreciate the reviewer's request for greater methodological specificity, and we want to respond with transparency about the reasons why the paper deliberately operates at the level of theoretical framing rather than prescriptive implementation. First, there is as yet no general consensus about what education is ultimately for - the lacuna that, as we argue throughout, is itself part of the problem AI forces into view. Without such consensus, any specific implementation framework risks encoding particular assumptions about educational aims that may be contested, culturally specific, or simply premature. We would further note that most existing educational environments are more restrictive than the principles we believe should guide schooling: agency, inquiry-based learning, collaborative and democratic practice, intrinsic motivation, and transparent evaluation, among others. Under these conditions, AI risks exacerbating already embedded contradictions rather than resolving them, making visible tensions that were previously absorbed by institutional inertia. Second, socio-economic disparities are likely to shape the effects of AI in education more powerfully than any specific design choice. Even if AI tools were universally available tomorrow, the evidence on educational inequality suggests that students from better-resourced family backgrounds would rapidly develop more sophisticated uses of these systems than their less advantaged peers, consistent with broader patterns of how schooling itself reproduces rather than reduces inequality. Any implementation framework that does not foreground this risk would be incomplete. Third,

meaningful transformation of educational environments is (mostly) a physical problem. The spatial arrangements of school buildings (e.g., the choice and positioning of classrooms, furniture, administrative offices, and communal spaces) reflect what the late Richard Elmore defined as a design of "control" that is deeply embedded in the material infrastructure of public education. These constraints cannot be wished away, and prescribing specific AI implementations or educational restructurations without acknowledging them would risk producing recommendations that are formally coherent but practically inert. For these reasons, we chose to offer a theoretical reframing that might travel across different institutional contexts rather than specify an implementation programme tied to conditions that vary enormously across schools, systems, and societies. We hope that this framing provides a more durable and adaptable foundation for future empirical and design work.

In conclusion, I congratulate the authors on their well-crafted article. It is dense, complex, and thought-provoking. Reading it has given me an original perspective on the problem of the impact of AI on schooling.

Dear Reviewer, we are genuinely grateful for this comment. As researchers who try to look at education from a bird's-eye view, this is precisely the kind of response we hoped our work would provoke. Our aim is not to redistribute educational labour, but to step back and consider the educational system as a whole, and to think carefully about what we can build, together, for future generations.
